# Human urinary kallidinogenase or edaravone combined with butylphthalide in the treatment of acute ischemic stroke

**DOI:** 10.1002/brb3.1438

**Published:** 2019-10-22

**Authors:** Yun Qian, Yi Lyu, Minhai Jiang, Bo Tang, Tian Nie, Shan Lu

**Affiliations:** ^1^ Department of Neurology Affiliated Hangzhou First People's Hospital Zhejiang University School of Medicine Hangzhou China; ^2^ Department of Anesthesiology Minhang Hospital Fudan University Shanghai China

**Keywords:** acute ischemic stroke, butylphthalide, edaravone, human urinary kallidinogenase

## Abstract

**Aim:**

The effectiveness of neuroprotective agents is still unclear. Here we analyzed the clinical outcomes of acute ischemic stroke (AIS) patients treated with human urinary kallidinogenase (HUK) or edaravone (Eda) combined with butylphthalide (NBP).

**Methods:**

From January 2016 to December 2017, a total of 165 AIS patients were enrolled in this open‐label, randomized controlled clinical study. Patients were randomly allocated into HUK group and Eda group in a ratio of 2:1. All the patients received basic treatments and NBP (200 mg p.o. qid) while HUK group received 0.15 PNA unit of HUK injection (ivgtt. qd) and Eda group received 30 mg Eda (ivgtt. bid) for 14 consecutive days. Independency rate [12‐month modified Rankin Scale (mRS) score ≤ 1] and related factors were compared between the two groups.

**Results:**

Twelve‐month mRS score of the HUK group (1, IQR 0~1) was significantly lower compared with Eda group (2, IQR 1~3, *p* < .0001). The HUK treatment achieved an independency rate of 79.1% while the Eda treatment only had 45.3% (*p* < .0001). Further binary logistic regression showed that recurrent stroke (RR: 0.1, 95% CI: 0.0~0.1, *p* = .038) and HUK treatment (RR: 4.2, 95% CI: 1.1~16.5, *p* = .041) could significantly affect patients' 12‐month outcomes.

**Conclusion:**

Human urinary kallidinogenase combined with NBP can enhance AIS patients' long‐term independency rate, and the effectiveness of HUK combined therapy is better than Eda.

## Graphical abstract

HUK combined with NBP can enhance AIS patients' long‐term independency rate, and the effectiveness of HUK combined therapy is better than Eda.

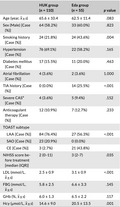

## 1 INTRODUCTION

The 2018 AHA/ASA guidelines for the early management of patients with acute ischemic stroke (AIS) have aroused much attention. Compared with the 2013 guidelines, several major changes based on new high‐quality evidence, including prehospital care, intravenous and intra‐arterial therapies, and in‐hospital management, have been made. One of the major changes is in the “General Supportive Care and Emergency Treatment” part that neuroprotective agents are not recommended because “at present, no pharmacological or nonpharmacological treatments with putative neuroprotective actions have demonstrated efficacy in improving outcomes after ischemic stroke” (Powers et al., 2018). However in real clinical practice, some neuroprotective agents indeed show their effectiveness in reducing oxidative stress, dilating arterioles in the ischemic area, and promoting patients' recovery. To AIS patients who are not eligible to IV thrombolytics or mechanical thrombectomy, neuroprotective agents do play an important and irreplaceable role. Hence in 2018 Chinese AIS diagnosis and treatment guidelines, individualized application of butylphthalide (NBP) and human urinary kallidinogenase (HUK) therapy can be taken into consideration according to the results of some randomized controlled trials (level IIb) (Chinese Society of Neurology, 2018).

NBP was approved for ischemic stroke by the State Food and Drug Administration in China in 2002 for the potential of inhibiting platelet aggregation (Peng, Zeng, Feng, & Wang, 2004), reducing ischemia‐induced oxidative damage (Li et al., 2009), improving microcirculation (Liu et al., 2007), attenuating mitochondrial dysfunction (Chen et al., 2018), and reconstructing the impaired neuronal network (Zhao, Liu, Li, Zhang, & Wang, 2019). In a Chinese randomized, double‐blind trial of 573 ischemic stroke patients, NBP treatment (both intravenous and oral) was safe and could improve outcomes at the third month after stroke (Cui et al., 2013).

The kallikrein–kinin system (KKS) is a complex endogenous enzyme system taking part in the regulation of heart disease, kidney disease, inflammation, cancer, and many other diseases (Chao & Chao, 2006; Emanuelia & Madeddu, 2003). Recently, it is also proved to protect against ischemic stroke (Chen et al., 2010; Zhang, Tao, Liu, & Wang, 2012). HUK can selectively dilate arterioles in the ischemic area (Nagano, Suzuki, Hayashi, & Asano, 1992), enhancing angiogenesis and neurogenesis (Stone et al., 2009), increasing regional cerebral blood flow, inhibiting apoptosis and inflammation (Xia et al., 2006), promoting glial cell migration (Lu et al., 2008), and improving neurological deficits after AIS (Ling et al., 2008). Meanwhile, edaravone (Eda) was first approved by the Japanese Ministry of Health in 2001 for the treatment of ischemic stroke. It scavenges free radicals (Higashi, Jitsuiki, Chayama, & Yoshizumi, 2006), inhibits lipid peroxidation and oxidative damage to brain cells, endothelial cells, and nerve cells (Yoshida et al., 2006), and reduces the effects of cerebral ischemia and edema (Nakamura et al., 2008), thus decreasing the tissue damage caused by acute cerebral infarction.

Since HUK ($71.5 per day) and Eda ($53.6 per day) are expensive in China, usually patients can only choose one drug in combination with NBP ($7.5 per day). Up till now, there is still no convincing evidence so we conducted this study to compare the effectiveness of HUK and Eda combined with NBP in the treatment of AIS, aiming to help healthcare givers and patients make cost‐effective decisions.

## 2 METHODS

### 2.1 Patients

Patients aged 18–85 years who were diagnosed with AIS were enrolled between January 2016 and December 2017 in the department of neurology at Affiliated Hangzhou First People's Hospital, Zhejiang University School of Medicine. Detailed inclusion criteria were as follows: (a) age ranging from 18 to 85 years; (b) AIS fitting the diagnostic criteria of cerebral infarction approved by the fourth national cerebrovascular academic conference (1995) (Chinese Neuroscience Society, Chinese Neurosurgical Society, 1996) and confirmed by head CT or MRI; and (c) with the first onset and onset time less than 48 hr. Exclusion criteria included: (a) bleeding disorder or bleeding trends in latest one month; (b) incomplete hepatic and renal function; (c) medical history of peptic ulcer, hemorrhagic stroke, brain tumor, and brain trauma; (d) patients who were eligible to IV thrombolytics or mechanical thrombectomy; (e) patients whose pertinent data could not be evaluated at the time of stroke onset; or (f) incomplete follow‐up or noncompliance with the study treatment.

The stroke subtypes were defined according to the Trial of Org 10172 in Acute Stroke Treatment (TOAST) classification system (Bamford, Sandercock, Dennis, Warlow, & Burn, 1991). The degree of carotid artery stenosis (CAS) was performed by radiologists who had more than 10 years of vascular ultrasonography experience using Philips IU 22 (Philips Healthcare), and severe stenosis was defined as 70%~99% according to the North American Symptomatic Carotid Endarterectomy Trial (NASCET) (Ferguson et al., 1999).

This study was reviewed and approved by the Ethics Committee at the Affiliated Hangzhou First People's Hospital, Zhejiang University School of Medicine. Written informed consent was obtained from each participant before enrollment.

### 2.2 Treatments

In this open‐label study, the 165 study subjects were randomly allocated in a 2:1 ratio into two treatment groups with random allocation sequence determined by a computer‐generated randomization chart. The 14‐day HUK group received 0.15 PNA unit of HUK injection (Trade name: Kailikang, Guangdong Techpool Bio‐Pharma Co.) in intravenous infusion, once a day, while the Eda group received 30 mg Eda (Trade name: Radicut, Mitsubishi Tanabe Pharma Corporation) in intravenous infusion, twice a day. All the patients received 200 mg NBP (Shijiazhuang Pharma Group) orally four times a day and basic treatment including antithrombosis, blood pressure and blood glucose control, statins, and dehydrating agents according to disease condition.

### 2.3 Outcome measurements

The primary outcome in this study was 12‐month independency rates of HUK therapy compared with Eda therapy using the modified Rankin Scale (mRS). Twelve‐month mRS score ≤ 1 was defined as independency (Gocmen, Arsava, Oguz, & Topcuoglu, 2018). Secondary outcomes were (a) nosocomial infection rates; (b) 12‐month recurrent stroke rates; and (c) factors related to the independency rate. Twelve‐month mRS scores were obtained by telephone follow‐up.

### 2.4 Statistical analysis

Statistical analysis was performed using the software IBM SPSS Statistics v.19 (SPSS Inc.). Baseline characteristics of the study patients were analyzed using chi‐square or Fisher's exact test as appropriate for categorical variables and Student's *t* test or Mann–Whitney test for continuous variables. Categorical variables were reported as number or percentage; continuous variables fitting the normal distribution were expressed as mean ± standard deviation (*SD*), whereas median (1st to 3rd quartile, interquartile ratio) was used for nonfitting variables. In addition, binary logistic regression analysis was performed to assess factors affecting 12‐month independency rate with feed‐forward model. A *p*‐value <.05 was considered statistically significant.

## 3 RESULTS

### 3.1 Baseline characteristics of all the patients

The baseline clinical and laboratory characteristics of the study patients are summarized in Table 1. Overall, there were no statistically significant differences in patient clinical characteristics between the two study groups except that more patients had smoking history (43.6% vs. 21.8%, *p* = .004) and transient ischemic attack (TIA) history (25.5% vs. 0, *p* < .0001) in Eda group. Patients' TOAST subtypes were not quite the same in our study: 20.9% of the HUK group was small‐artery occlusion (SAO) while nearly a half (43.8%) of patients in the Eda group was cardioembolism (CE, *p* < .0001). Moreover, the National Institute of Health stroke scale (NIHSS) scores before treatment were 2 (IQR 0–11) and 3 (IQR 2–7) for the HUK and Eda groups, respectively (*p* = .035), and the two groups differed significantly in low‐density lipoprotein (LDL, 2.5 ± 0.9 vs. 3.1 ± 0.9, *p* < .0001) and homocysteine (Hcy, 14.6 ± 9.0 vs. 20.5 ± 13.5, *p* = .0001) concentration.

**Table 1:** Patients' basic characteristics

	HUK group (*n* = 110)	Eda group (*n* = 55)	*p* value
Age (year, $\bar{x}\pm s$)	65.6 ± 10.4	62.5 ± 11.4	.083
Sex (Male) [Case (%)]	64 (58.2%)	33 (60.0%)	.823
Smoking history [Case (%)]	24 (21.8%)	24 (43.6%)	.004
Hypertension [Case (%)]	76 (69.1%)	22 (58.2%)	.165
Diabetes mellitus [Case (%)]	17 (15.5%)	11 (20.0%)	.463
Atrial fibrillation [Case (%)]	4 (3.6%)	2 (3.6%)	1.000
TIA history [Case (%)]	0 (0.0%)	14 (25.5%)	<.001
Severe CAS^a^ [Case (%)]	4 (3.6%)	5 (9.4%)	.152
Anticoagulant therapy [Case (%)]	12 (10.9%)	7 (12.7%)	.233
TOAST subtype			
LAA [Case (%)]	84 (76.4%)	27 (56.3%)	<.001
SAO [Case (%)]	23 (20.9%)	0 (0.0%)	
CE [Case (%)]	3 (2.7%)	21 (43.8%)	
NIHSS score before treatment [median (IQR)]	2 (0–11)	3 (2–7)	.035
LDL (mmol/L, $\bar{x}\pm s$)	2.5 ± 0.9	3.1 ± 0.9	<.001
FBG (mmol/L, $\bar{x}\pm s$)	5.8 ± 2.5	6.6 ± 3.3	.145
GHb (%, $\bar{x}\pm s$)	6.0 ± 1.3	6.5 ± 2.2	.117
Hcy (μmol/L, $\bar{x}\pm s$)	14.6 ± 9.0	20.5 ± 13.5	.001

Note

CAS, carotid artery stenosis; CE, cardioembolism; FBG, fasting blood glucose; GHb, glycosylated hemoglobin; Hcy, homocysteine; LAA, large‐artery atherosclerosis; LDL, low‐density lipoprotein; NIHSS, National Institute of Health stroke scale; SAO, small‐artery occlusion; TIA, transient ischemic attack.

a Severe CAS = 70~99% carotid artery stenosis.

Seven patients (6.4%) in the HUK group and 5 (9.1%) in the Eda group accepted anticoagulant therapy (*p* = .537) based on their disease severity and eligibility.

### 3.2 The primary outcome

Twelve‐month mRS score of the HUK group (1, IQR 0~1) was significantly lower compared with Eda group (2, IQR 1~3, *p* < .0001). The HUK treatment achieved an independency rate of 79.1% while the Eda treatment only had 45.3% (*p* < .0001, Table 2).

**Table 2:** Patients' outcomes of two groups

	HUK group (*n* = 110)	Eda group (*n* = 55)	*p* value
12‐month mRS [median (IQR)]	1 (0–1)	2 (1–3)	<.001
Nosocomial infection [Case (%)]	2 (1.8%)	1 (1.8%)	1.000
Recurrent stroke [Case (%)]	5 (4.6%)	5 (9.4%)	.299

Note

mRS, modified Rankin Scale.

### 3.3 Secondary outcomes

No significant difference was found in nosocomial infection rates and 12‐month recurrent stroke rates between the two groups (Table 2). Binary logistic regression showed that HUK treatment could lead to a 4.2‐fold higher rate of patients' independency 12 months after AIS occurrence (RR: 4.2, 95% CI: 1.1~16.5, *p* = .041) while recurrent stroke in 12 months could reduce patients' independency rate with 0.1‐fold (RR: 0.1, 95% CI: 0.0~0.1, *p* = .038, Table 3).

**Table 3:** Logistic analysis of factors related to patients' 12‐month independency rate

Risk factors	OR value	95% CI	*p* value	Adjusted OR value	95% CI	*p* value
Female	1.1	0.6~2.1	.813			
Age	1.0	0.9~1.0	.975			
Smoking history	1.0	0.5~2.0	.903			
Hypertension	0.8	0.4~1.6	.510			
Diabetes mellitus	0.6	0.3~1.5	.283			
Atrial fibrillation	2.4	0.3~21.1	.428			
TIA history	0.1	0.0~0.4	.002	0.5	0.1~2.9	.408
Severe CAS^a^	0.4	0.1~1.8	.264			
NIHSS score before treatment	0.8	0.8~0.9	<.001	0.8	0.7~0.9	.769
TOAST subtype						
LAA	/	/	/	/	/	/
SAO	11.3	1.5~87.2	.020	6.1	0.7~52.2	.096
CE	0.9	0.3~2.1	.740	1.0	0.3~4.0	.944
LDL	0.8	0.5~1.1	.184			
FBG	0.9	0.8~1.0	.039	0.9	0.8~1.1	.341
GHb	1.0	0.8~1.2	.961			
Hcy	1.0	0.9~1.0	.131			
Nosocomial infection	0.2	0.0~2.6	.231			
Recurrent stroke	0.1	0.0~0.5	.005	0.1	0.0~0.1	.038
Anticoagulant therapy	0.6	0.2~2.1	.454			
HUK treatment	4.6	2.2~9.3	<.001	4.2	1.1~16.5	.041

Note

CAS, carotid artery stenosis; CE, cardioembolism; FBG, fasting blood glucose; GHb, glycosylated hemoglobin; Hcy, homocysteine; HUK, human urinary kallidinogenase; LAA, large‐artery atherosclerosis; LDL, low‐density lipoprotein; NIHSS, National Institute of Health stroke scale; SAO, small‐artery occlusion; TIA, transient ischemic attack.

a Severe CAS = 70%~99% carotid artery stenosis.

## 4 DISCUSSION

Butylphthalide has been widely used in Chinese AIS patients since its approval in 2002, and after that, several Chinese multicenter, open‐label clinical studies demonstrated its effectiveness and safety (Cui & Li, 2006; Cui et al., 2005, 2008). NBP has a variety of biological activities including suppressing platelet aggregation and thrombosis (Peng et al., 2004), improving cerebral microcirculation (Liu et al., 2007; Yan, Feng, & Zhang, 1998) and reducing ischemia‐induced oxidative damage (Li et al., 2009), all of which were associated with brain injury caused by ischemic stroke. However, the mechanism of NBP for the treatment of AIS is still unrevealed. One possible way was up‐regulation of vascular endothelial growth factor (VEGF) and hypoxia‐inducible factor (HIF)‐1 alpha expression, which was associated with angiogenesis (Cao, Deji, Li, He, & Zhou, 2009; Liao et al., 2009).

Acute ischemic stroke can lead to excessive Ca^2+^ influx and formation of reactive oxygen species (ROS), causing the death of neuronal cells due to mitochondrial dysfunction (Siesjö et al., 1999). Eda was shown to capture and reduce excessive ROS, thus preventing brain damage (Watanabe, Tanaka, Watanabe, Takamatsu, & Tobe, 2004). In 2003, Eda Acute Infarction Group reported that 30 mg Eda‐treated patients had significantly lower mRS, both short‐term and long‐term (Edaravone Acute Infarction Study Group, 2003). Another retrospective study in 70 patients with lacunar infarction investigated patients who received 30 mg Eda twice daily for 14 days and found a significant odds ratio effect in patients that received Eda treatment (Mishina et al., 2005).

Like Eda, HUK was reported to suppress oxidative stress through tissue KKS (Chao & Chao, 2005). And like NBP, HUK could induce the expression of VEGF, leading to new blood vessels formation by transfer of kinins (Emanueli & Madeddu, 2004). Furthermore, kinin and kallidin, which were transferred from kininogen hydrolysis by HUK, could combine with bradykinin B1 receptor produced under induction of ischemic brain tissue to release NO and relax vascular smooth muscle (Lagneux, Adam, & Lamontagne, 2003; Li et al., 2008; Sangsree, Brovkovych, Minshall, & Skidgel, 2003). Thus, it can selectively dilate arterioles in the ischemic area and increase regional cerebral blood flow. In 2010, a systematic review on HUK's efficacy and safety in stroke studied 24 trials and demonstrated that 22 trials with 2,117 patients benefited remarkably from HUK treatment (Zhang et al., 2012). Compared with Eda, HUK takes part in much more physiological activities, and that can partly explain our study results that HUK combined with NBP raises AIS patients' independency rate better than Eda plus NBP.

Although there were some differences in patients' basic characteristics between the two groups, such as smoking history, TOAST subtypes, and NIHSS score before treatment, through binary logistic regression, all these factors would not affect patients' independency rate. Besides, with all the risk factors being eliminated, HUK treatment alone could raise patients' independency rate of 4.2‐fold higher.

Pharmaceutical companies never give up exploring novel neuroprotective agents. For example, compound 10b, a hybrid of Eda and a ring‐opening derivative of NBP, has recently been reported to significantly reduce the infarct volume and neurological deficits in rats, and its neuroprotective effects have been more pronounced compared with NBP, Eda, or NBP + Eda (Hua et al., 2015). Since the reason why neuroprotective agents were not recommended by 2018 AHA/ASA guidelines was that no neuroprotective agent met the quality of evidence, we should conduct more large‐scale clinical trials to confirm the safety and efficacy of neuroprotective agents instead of stop using them.

## 5 CONCLUSION

Human urinary kallidinogenase combined with NBP can enhance AIS patients' long‐term independency rate, and the effectiveness of HUK combined therapy is better than Eda. HUK combined with NBP may be a more cost‐effective treatment for AIS patients.

## CONFLICT OF INTEREST

None declared.

## Data Availability

All original data will be available when contact the correspondence author by email.
